# Effect of *iclR *and *arcA *knockouts on biomass formation and metabolic fluxes in *Escherichia coli *K12 and its implications on understanding the metabolism of *Escherichia coli *BL21 (DE3)

**DOI:** 10.1186/1471-2180-11-70

**Published:** 2011-04-11

**Authors:** Hendrik Waegeman, Joeri Beauprez, Helena Moens, Jo Maertens, Marjan De Mey, Maria R Foulquié-Moreno, Joseph J Heijnen, Daniel Charlier, Wim Soetaert

**Affiliations:** 1 Centre of Expertise-Industrial Biotechnology and Biocatalysis, Department of Biochemical and Microbial Technology, Ghent University, Coupure Links 653, B-9000 Ghent, Belgium; 2 Laboratory for Genetics and Microbiology, Department of Applied Biological Sciences, Vrije Universiteit Brussel, Pleinlaan 2, B-1050 Brussels, Belgium; 3 Kluyver Laboratory for Biotechnology, Department of Biochemical Engineering, Delft University of Technology Julianalaan 67, 2628 BC Delft

## Abstract

**Background:**

Gene expression is regulated through a complex interplay of different transcription factors (TFs) which can enhance or inhibit gene transcription. ArcA is a global regulator that regulates genes involved in different metabolic pathways, while IclR as a local regulator, controls the transcription of the glyoxylate pathway genes of the *aceBAK *operon. This study investigates the physiological and metabolic consequences of *arcA *and *iclR *deletions on *E. coli *K12 MG1655 under glucose abundant and limiting conditions and compares the results with the metabolic characteristics of *E. coli *BL21 (DE3).

**Results:**

The deletion of *arcA *and *iclR *results in an increase in the biomass yield both under glucose abundant and limiting conditions, approaching the maximum theoretical yield of 0.65 c-mole/c-mole glucose under glucose abundant conditions. This can be explained by the lower flux through several CO_2 _producing pathways in the *E. coli *K12 Δ*arcA*Δ*iclR *double knockout strain. Due to *iclR *gene deletion, the glyoxylate pathway is activated resulting in a redirection of 30% of the isocitrate molecules directly to succinate and malate without CO_2 _production. Furthermore, a higher flux at the entrance of the TCA was noticed due to *arcA *gene deletion, resulting in a reduced production of acetate and less carbon loss. Under glucose limiting conditions the flux through the glyoxylate pathway is further increased in the Δ*iclR *knockout strain, but this effect was not observed in the double knockout strain. Also a striking correlation between the glyoxylate flux data and the isocitrate lyase activity was observed for almost all strains and under both growth conditions, illustrating the transcriptional control of this pathway. Finally, similar central metabolic fluxes were observed in *E. coli *K12 Δ*arcA *Δ*iclR *compared to the industrially relevant *E. coli *BL21 (DE3), especially with respect to the pentose pathway, the glyoxylate pathway, and the TCA fluxes. In addition, a comparison of the genome sequences of the two strains showed that BL21 possesses two mutations in the promoter region of *iclR *and rare codons are present in *arcA *implying a lower tRNA acceptance. Both phenomena presumably result in a reduced ArcA and IclR synthesis in BL21, which contributes to the similar physiology as observed in *E. coli *K12 Δ*arcA*Δ*iclR*.

**Conclusions:**

The deletion of *arcA *results in a decrease of repression on transcription of TCA cycle genes under glucose abundant conditions, without significantly affecting the glyoxylate pathway activity. IclR clearly represses transcription of glyoxylate pathway genes under glucose abundance, a condition in which Crp activation is absent. Under glucose limitation, Crp is responsible for the high glyoxylate flux, but IclR still represses transcription. Finally, in *E. coli *BL21 (DE3), ArcA and IclR are poorly expressed, explaining the similar fluxes observed compared to the Δ*arcA*Δ*iclR *strain.

## Background

The genome of the bacterium *Escherichia coli *consists of 4.6 million base pairs and contains 4288 genes [1]. If all genes would be transcribed simultaneously, the cell volume should be at least threefold higher to harbor all proteins produced. Furthermore, under specific environmental conditions, transcription of only a limited set of genes is necessary to ensure optimal growth. In order to control which genes are transcribed, transcription is controlled by the interplay of numerous regulators [2].

Transcriptional regulators or transcription factors (TFs) are proteins that bind to specific sequences of the DNA, *i.e. *promoters, and hereby facilitate or inhibit the binding of RNA polymerase (RNAP). A low RNAP affinity generally results in low gene expression, while a higher RNAP affinity corresponds with an increased gene expression. However, if the affinity is too strong, gene expression decreases again due to a too weak mobility of the RNAP [3-5].

Regulation of gene expression is very complex and transcriptional regulators can be subdivided into global and local regulators depending on the number of operons they control. Global regulators control a vast number of genes, which must be physically separated on the genome and belong to different metabolic pathways [6]. Only seven global regulators are required to control the expression of 51% of all genes: ArcA, Crp, Fis, Fnr, Ihf, Lrp, and NarL. In contrast to global regulators, local regulators control only a few genes, *e.g. *20% of all TFs control the expression of only one or two genes [7]. The regulators investigated in this study are the global regulator ArcA and the local regulator IclR.

ArcA (anaerobic redox control) was first discovered in 1988 by Iuchi and Lin and the regulator seemed to have an inhibitory effect on expression of aerobic TCA cycle genes under anaerobic conditions [8]. ArcA is the regulatory protein of the dual-component regulator ArcAB, in which the later discovered ArcB acts as sensory protein [9]. Statistical analysis of gene expression data [10] showed that ArcA regulates the expression of a wide variety of genes involved in the biosynthesis of small and macromolecules, transport, carbon and energy metabolism, cell structure, *etc*. The regulatory activity of ArcA is dependent on the oxygen concentration in the environment and the most profound effects of *arcA *gene deletion are noticed under microaerobic conditions [11]. In contrast, under anaerobic conditions Fnr (fumarate nitrate reductase) is the predominant redox sensing global regulator [12-14]. Recently however, it was discovered that also under aerobic conditions ArcA has an effect on central metabolic fluxes [15].

The second regulator investigated in this study, isocitrate lyase regulator (IclR), represses the expression of the *aceBAK *operon, which codes for the glyoxylate pathway enzymes isocitrate lyase (AceA), malate synthase (AceB), and isocitrate dehydrogenase kinase/phosphatase (AceK) [16]. The last enzyme phosphorylates the TCA cycle enzyme isocitrate dehydrogenase (Icd), which results in a reduction of Icd activity and consequently in a reduction of the flux through the TCA cycle [17]. When IclR levels are low or when IclR is inactivated, *i.e. *for cells growing on acetate [18-20], or in slow-growing glucose-utilizing cultures [21,22], repression on glyoxylate genes is released and the glyoxylate pathway is activated.

Although the effect of single deletions of genes, coding for global regulators, on metabolism have been extensively studied [15,23], their double knockouts have rarely been investigated. So far, *in vivo *only the effects of *arcA-fnr *[12], *arcA-cra *[24], and *crp-fur *[25] knockout combinations have been studied. Recently, two studies focused on the effect of the deletion of genes coding for a global regulator and a local regulator, *i.e. cra-iclR *and *crp-iclR *[26,27], on gene expression and activities of key metabolic enzymes. However, the effect of the knockouts on the metabolic fluxes were not investigated.

This study investigates such a knockout combination and shows that the combined deletion of *arcA *and *iclR *has a profound effect on metabolism and redirects carbon fluxes in such a way that the biomass content increases remarkably both under glucose abundant and glucose limiting conditions as opposed to its parent strain *E. coli *K12 MG1655. Many of the observed characteristics in the double knockout strain are also ascribed to *E. coli *BL21 (DE3), which is why fluxes between these two strains were investigated as well.

## Results and Discussion

### Physiological effects of *arcA *and *iclR *deletions

**Figure 1:**
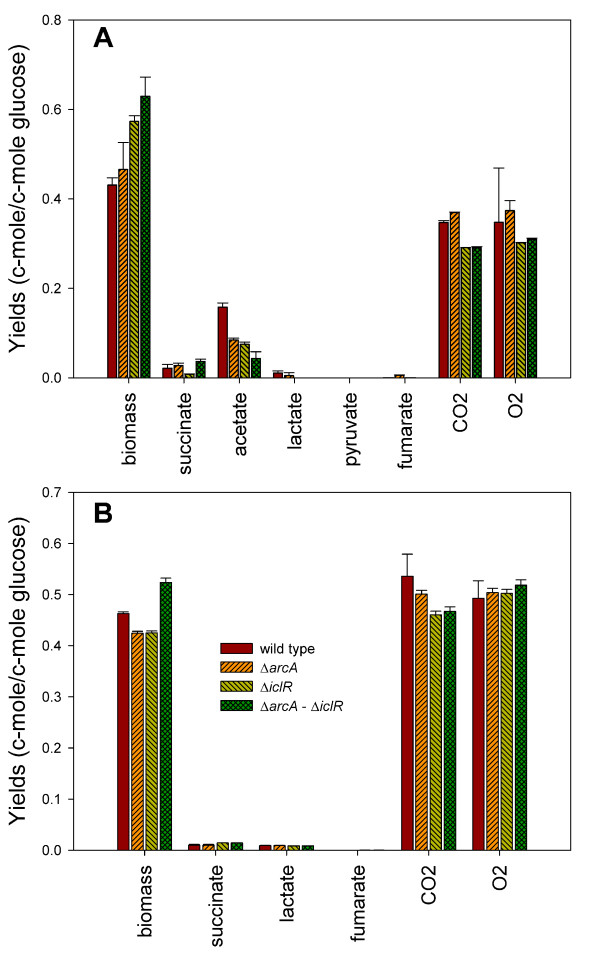
**Product yields of the wild type and knockout strains**. Product yields in c-mole/c-mole glucose of the wild type MG1655, the derived single knockout strains Δ*arcA *and Δ*iclR*, and the double knockout strain Δ*arcA*Δ*iclR *under glucose abundant, batch (A) and glucose limiting, chemostat (B) conditions. Oxygen yield is shown as a positive number for a clear representation, but *O*_2 _is actually consumed during the experiments. The values represented in the graph are the average of at least two separate experiments and the errors are standard deviations calculated on the yields.

Wild type MG1655, single and double knockout strains were first cultivated in a 2*L *bioreactor under glucose abundant (batch) and limiting (chemostat, *D *= ±0.1 *h *^-1^) conditions in order to precisely determine extracellular fluxes and growth rates. The growth rates are shown in Table 1. The *arcA *and *iclR *single knockout strains have a slightly lower maximum growth rate. The *arcA*-*iclR *double knockout strain exhibits a reduction of as much as 38% in *μ*_max_. Figure 1 shows the effects of these mutations on various product yields under batch and chemostat conditions for the different strains. The corresponding average redox and carbon balances close very well (data shown in Additional file 1). The phenotypic effects will be discussed below.

**Table 1:** Average maximum growth rates (batch) and dilution rates (chemostat) of the different strains.

	Batch	Chemostat	
**Strain**	***μ_max_*(*h*^-1^)**	***D_influent_*(*h*^-1^)**	***D_effluent_*(*h*^-1^)**

Wild type	0.66 ± 0.02	0.099 ± 0.001	0.100 ± 0.001
Δ*arcA*	0.60 ± 0.01	0.118 ± 0.001	0.120 ± 0.001
Δ*iclR*	0.61 ± 0.02	0.085 ± 0.001	0.090 ± 0.001
Δ*arcA*Δ*iclR*	0.44 ± 0.03	0.090 ± 0.001	0.093 ± 0.001

Under chemostat conditions, the apparent growth rate equals the dilution rate of the influent. Differences between *D_influent _*and *D_effluent _*are due to addition of base and acid for pH correction and sampling.

Under glucose abundant conditions (see Figure 1A), the following trends can be observed. Both the *arcA *and *iclR *knockout strains show an increased biomass yield. When combining these deletions (*i.e. *in Δ*arcA*Δ*iclR*) the yield is further increased to 0.63 ± 0.01 c-mole/c-mole glucose, which approximates the theoretical biomass yield of 0.65 c-mole/c-mole glucose (assuming a P/O-ratio of 1.4) [28,29]. The higher biomass yield is accompanied by a 70 and 16% reduction in acetate and CO_2_, respectively.

The results of the glucose limited cultures are shown in Figure 1B. The Δ*arcA*Δ*iclR *strain exhibits an increased biomass yield compared to the wild type strain (0.52 ± 0.01 c-mole/c-mole vs. 0.46 ± 0.01 c-mole/c-mole), but the increment in biomass yield (*i.e. *13%) is less distinct as observed under glucose abundant conditions (47%).

The increment in biomass yield is less pronounced under glucose limitation, because glucose limited cultures of the strain Δ*arcA*Δ*iclR *show a decreased biomass yield while the wild type shows an increased biomass yield compared to if these strains are cultivated under glucose abundant conditions. This can be easily explained: under glucose abundance, the wild type strain converts 16% of the carbon source to acetate as a result of overflow metabolism [30]. At a fixed, low growth rate and consequently under glucose limitation, the cell can easily cope with the delivered carbon and very little carbon is dissipated through formation of byproducts. However, energy losses also occur in continuous cultures because of the existence of futile cycles [31]. In addition, as shown by Pirt and many others, an excessive fraction of the energy source is reserved for growth-independent maintenance, a factor which is relatively higher under glucose limitation [32-36]. For the wild type cultivated at a low growth rate (*D *= ±0.1 *h*^-1^), the absence of energy spilling by overflow metabolism compensates and even exceeds the energy spilling by futile cycling and the energy reserved for maintenance, explaining the higher biomass yield observed. In contrast, the Δ*arcA *Δ*iclR *strain does not show overflow metabolism under glucose abundance, and therefore the effects of energy loss by futile cycles and maintenance are more visible in this strain leading to a lower biomass yield under glucose limitation.

**Figure 2:**
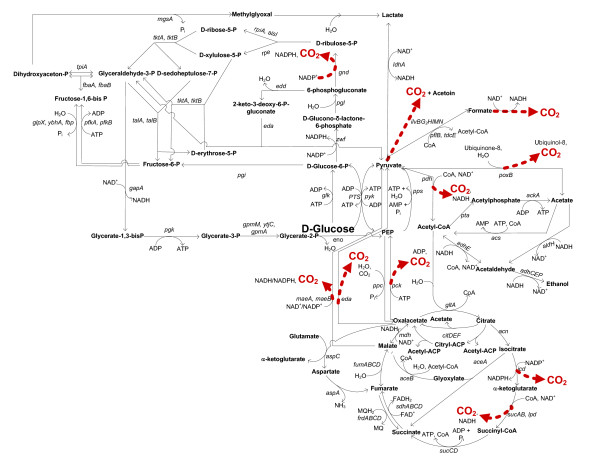
***Escherichia coli *central metabolism**. CO_2 _forming reactions are emphasized. Genes coding for corresponding metabolic enzymes are shown in italic. The genes and their gene products are listed in Additional file 2.

For all experiments in which significantly higher biomass yields were observed, *i.e. *for Δ*iclR *in glucose abundant conditions and for Δ*arcA*Δ*iclR *in glucose abundant and limiting conditions, the high yield is linked to a reduction in CO_2 _yield. In Figure 2 all CO_2 _forming reactions located in central metabolism are emphasized (corresponding gene products are shown in Additional file 2): the oxidative part of the TCA cycle, the entrance of the pentose phosphate pathway, the gluconeogenic reactions from malate and OAA to pyruvate and the fermentative reactions from pyruvate to acetate, formate, and acetoïne. Since ArcA and IclR repress expression from the *aceBAK *operon, it is likely that the glyoxylate pathway, which is a parallel pathway of the TCA cycle but does not lead to CO_2 _production, is active in the double knockout strain. Consequently, the activity of glyoxylate enzymes and central metabolic fluxes of the four strains were determined.

### Activity of glyoxylate cycle enzymes

**Figure 3:**
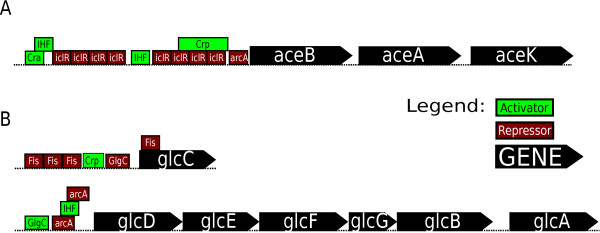
**Transcriptional regulation of the *aceBAK *and the *glc *operon**. (A): the *aceBAK *operon. Genes encode for the following enzymes; *aceB*: malate synthase A, *aceA*: isocitrate lyase, *aceK*: isocitrate dehydrogenase kinase/phosphatase. IclR and ArcA are repressors, FruR and IHF activate transcription [57]. The role of Crp is somewhat unclear. It has been reported as a repressor [25,39], but metabolic flux analysis and enzyme activity measurements show its role as an activator [23,83]. (B): the *glc *operons. Genes encode for the following enzymes; *glcC*: glycolate DNA binding regulator, *glcDEF*: glycolate oxidase subunits, *glcG*: conserved protein with unknown function, *glcB*: malate synthase G, *glcA*: glycolate transporter. ArcA and Fis are transcriptional repressors, Crp and IHF are activators. GlgC (glucose-1-phosphate adenylyltransferase, active in glycogen biosynthesis) activates the *glcD *operon and represses the *glcC *operon [57].

If the glyoxylate shunt is active in the Δ*arcA*Δ*iclR *strain, enzyme levels of the pathway should be upregulated. In Table 2 the relative enzyme activities of isocitrate lyase and malate synthase are depicted. The corresponding reactions are denoted in Figure 2 by the gene names *aceA *and *aceB*, respectively. ArcA and IclR are known regulators of the *aceBAK *operon and their regulatory recognition sites in the promoter region are illustrated in Figure 3A. The results of both enzyme activity measurements will be discussed below.

**Table 2:** Relative activities of malate synthase and isocitrate lyase under glucose abundant (batch) and limiting (chemostat) conditions.

	Isocitrate lyase activity		Malate synthase activity	
**Strain**	**Batch**	**Chemostat**	**Batch**	**Chemostat**

MG1655	1.00 ± 0.10	10.13 ± 1.43	1.00 ± 0.19	0.11 ± 0.03
MG1655 Δ*arcA*	0.33 ± 0.04	32.47 ± 3.61	0.36 ± 0.07	2.13 ± 0.39
MG1655 Δ*iclR*	5.69 ± 0.57	26.96 ± 3.06	1.38 ± 0.27	0.24 ± 0.04
MG1655 Δ*arcA*Δ*iclR*	6.39 ± 0.64	26.52 ± 2.78	0.48 ± 0.08	2.92 ± 0.52

Arbitrarily, all enzyme activities are scaled to the wild type activities under glucose abundant conditions.

The isocitrate lyase activity levels of the strains cultivated under glucose abundant conditions are rather low compared to those obtained under glucose limiting conditions. Remarkably, under glucose excess deletion of *iclR *results in an almost sixfold increase in the enzymes activity compared to the wild type. Deleting *arcA *as well (in the resulting Δ*arcA*Δ*iclR *strain) did not evoke a significant increase in isocitrate lyase activity compared to the enzyme activity in the Δ*iclR *strain, which indicates that IclR is a stronger repressor of the *aceBAK *operon as opposed to ArcA under these conditions.

Under glucose limiting conditions, the wild type isocitrate lyase activity is enhanced 10 times compared to batch conditions, which is in accordance with previous proteome analysis of glucose limited cultures [37,38] and enzyme activity levels [22,38] under similar growth conditions. This is presumably due to different cAMP levels under glucose abundant and limiting conditions, since cAMP binding to Crp is necessary for regulatory activity of Crp.

Under high glucose levels, cAMP concentrations are low and the cAMP-Crp complex cannot be formed. Consequently, activation of transcription of glyoxylate pathway genes by Crp cannot occur. If *crp *is deleted from the genome (*i.e. *in a Δ*crp *strain), no major differences in transcript levels of *aceA *or *aceB *between a culture grown under high and low glucose levels should be noticed, which was confirmed by transcriptome analysis [39]. Furthermore if Crp represses transcription of glyoxylate genes under high glucose levels as alleged in a few studies [25,39], a difference in *aceA *and *aceB *transcript levels should be noticed between the wild type and the *crp *knockout strain under high glucose concentrations, which was not observed [39].

Under glucose limiting conditions however, cAMP levels rise and the cAMP-Crp complex is properly formed, enabling the functioning of the regulator. Now Crp binds the DNA, competes with the binding of the repressor IclR and hereby activates transcription. If under these low glucose concentrations Crp is absent (*i.e. *in a Δ*crp *strain), the activities of the enzymes involved in the glyoxylate shunt should drop, since IclR can now fully repress *aceBAK *transcription. This was confirmed by Nanchen *et al. *who studied the behavior of a Δ*crp *strain under glucose limitation [23]. However, the transcription of glyxoylate genes is the result of the regulatory activity of multiple regulators and not only Crp. If the repressors IclR and ArcA are inactive, *i.e. *in the Δ*iclR *and the Δ*arcA *strain, isocitrate lyase levels are increased compared to the wild type (see Table 2).

The malate synthase activity in *E. coli *is the result of the activity of two isoenzymes, malate synthase A (gene: *aceB*) and G (gene: *glcB*) [40]. Both genes are members of different operons and the corresponding enzymes are members of different pathways, *i.e. *malate synthase A is the second enzyme of the glyoxylate pathway, whereas malate synthase G acts in the glycolate pathway. Figure 3B depicts the transcriptional regulation of the *glc *operons.

The obtained malate synthase activities (see Table 2) are somewhat contra-intuitive. Since Crp is also an activator of the *glcC *operon [41], one expects similar activity levels for isocitrate lyase and malate synthase, which was not observed. Nonetheless, some conclusions can be derived from the data. ArcA represses both *glcB *and *aceB *expression, thus both enzyme activities should increase in the knockout strain (assuming that there is no translational regulation involved). This explains the twentyfold increment in malate synthase activity in the Δ*arcA *strain under glucose limiting conditions. Rather small differences are noticed between the wild type and the Δ*iclR *strain in both growth conditions, implying that IclR does not greatly affect malate synthase activity. Either IclR has a moderate influence on gene expression of malate synthase A, or post-translational effects are taking place, or the malate synthase activity is primarily the result of the malate synthase G activity (*glcB*), as IclR is not a regulator of the *glc *operons. If IclR has a limited influence on *aceB *expression, one expects a similar action on *aceA *as both genes are members of the same operon. Second, if the activity is heavily affected by post-translational events, one does not expect such great differences between the Δ*arcA *strain and the wild type or ArcA should have an influence on the post-translational process. Since the former phenomena were not observed, it is very likely that the malate synthase activity is predominantly the result of *glcB *expression. Other regulators of the *glc *operon, besides ArcA and Crp, are GlcC, IHF, and Fis (Figure 3B). The action of these other regulators can explain the results of the batch cultures. The activator IHF has limited activity in exponentially growing cells [42], but the regulation of the *glc *operon is even further complicated by the possibility of acetate cross-inducing the operon [43].

Because of the interference of the malate synthase G activity in the measurement of malate synthase activity, it can be concluded that the measurement of isocitrate lyase activity is a better indicator for glyoxylate pathway activity.

### Glycogen and trehalose content

The aberrantly higher redox balance noticed in the Δ*arcA*Δ*iclR *strain (see Additional file 1) indicates that the biomass composition is slightly different in this strain. For example, as a reaction to unfavorable conditions, microorganisms can store certain polymers and fatty acids [44,45]. These compounds will increase the net weight of the biomass and will consequently alter the relative biomass composition. Thus, a measured higher biomass yield does not necessarily imply a higher biomass synthesis in terms of RNA, DNA, and protein. The two predominant molecules that *E. coli *can store under different environmental conditions are glycogen and trehalose [46-49] and therefore the contents of these compounds were determined in both the wild type and the Δ*arcA*Δ*iclR *strain under glucose abundance and glucose limitation. Trehalose was not detected in any of the cases. For both growth conditions, the glycogen content was higher in the double knockout strain compared to the wild type (see Table 3). However, the 1% increase in glycogen content cannot explain the sharp increase in biomass yield in the Δ*arcA*Δ*iclR *strain.

**Table 3:** Glycogen content of the wild type and the double knockout strain under glucose abundant (batch) and glucose limiting (chemostat) conditions.

Strain	Batch	Chemostat
MG1655	0.25 ± 0.26	0.50 ± 0.24
MG1655 Δ*arcA*Δ*iclR*	1.47 ± 0.19	1.29 ± 0.16

Values are expressed as carbon relative to the total amount of biomass carbon. The results shown are the averages of two cultures, measured 4 times. The wild type chemostat culture had a dilution rate of 0.17 ± 0.01 *h*^-^^1^; the Δ*arcA*Δ*iclR *strain had a dilution rate of 0.33 ± 0.02 *h*^-^^1^. The carbon balance and redox balance for these experiments are similar to the data shown in Additional file 1

Considering the product yield and storage compound results, it can be concluded that the increase in biomass yield in the double knockout strain is primarily the result of the lower acetate and CO_2 _production under glucose abundant conditions and of the lower CO_2 _production under glucose limitation. Only a small and similar amount of the extra carbon is converted to storage molecules like glycogen under both growth conditions.

### Effect of *arcA *and *iclR *knockouts on metabolic fluxes

**Figure 4:**
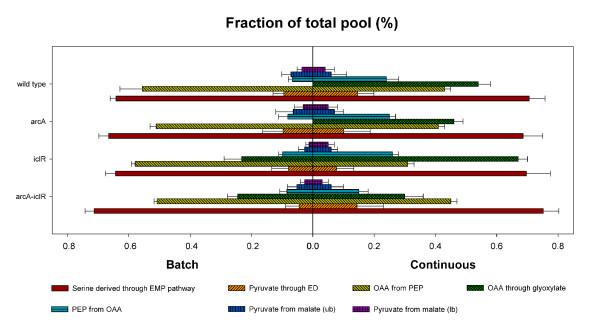
**Origin of metabolic intermediates in *E. coli *MG1655 single knockout strains Δ*arcA *and Δ*iclR*, and the double knockout strain Δ*arcA*Δ*iclR *cultivated in glucose abundant (batch) and glucose limiting (continuous) condtions**. Standard deviations are calculated on different samples originating from different cultivations. The serine through EMP and the pyruvate through ED results were obtained from experiments using 50% 1-^13^C glucose and 50% naturally labeled glucose. To determine the remaining values a mixture of 20% U-^13^C glucose and 80% naturally labeled glucose was used. To determine the fractions resulting in the formation of OAA a Monte-Carlo approach was applied. For chemostat experiments, a dilution rate of 0.1 *h*^-1 ^was set.

The arcA and *iclR *gene deletions have a profound effect on the phenotype of the resulting strains and on the activity of some key central metabolic enzymes under the different growth conditions as shown in the previous sections. In order to understand the metabolic implications of these deletions and consequently to grasp the role of IclR and ArcA in central metabolism, metabolic flux ratios and the corresponding net fluxes were determined. Figure 4 shows the origin of different intermediate metabolites of the different strains grown in batch and continuous mode.

Under glucose abundant conditions, deleting *arcA *results in a decrease of the OAA from PEP fraction, indicating that a higher fraction of OAA originates from the TCA cycle (OAA from TCA = 1 - OAA from PEP - OAA from glyoxylate). This phenomenon is also observed in the double knockout strain. Deletion of iclR results in an increase of the OAA from glyoxylate fraction from 0 to 23%. This effect is also retained in the double knockout strain Δ*arcA *Δ*iclR*. A third effect noticed in the double knockout strain is the significantly increased amount of serine originating from the Embden-Meyerhof-Parnas pathway (glycolysis) compared to the wild type (see Figure 4).

Under glucose limiting conditions a higher fraction of serine through EMP was observed for all strains as compared to the wild type under batch conditions. Furthermore the OAA from glyoxylate and the PEP from OAA fractions are increased compared to under glucose excess, implying the activation of the glyxylate cycle and gluconeogenesis. These fractions are even further increased in the Δ*iclR *strain which proves that also under glucose limiting conditions, IclR regulates the glyoxylate shunt, together with Crp and other regulators. In the double knockout strain the OAA from glyoxylate fraction decreases compared to the Δ*iclR *strain, which seems to be affected by the *arcA *deletion (see Figure 4). This is not expected as both IclR and ArcA are repressors of the pathway.

**Figure 5:**
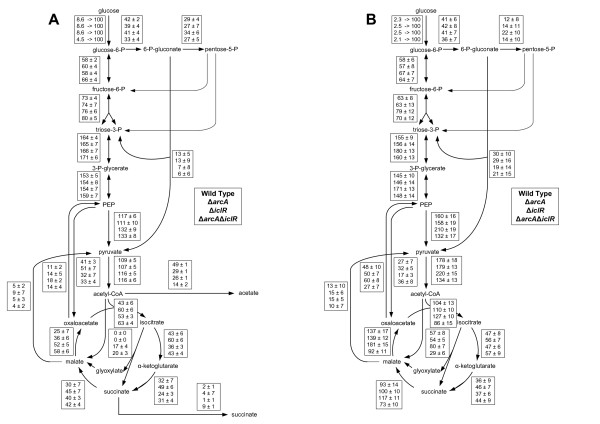
**Metabolic flux distribution in *E. coli *MG1655 single knockout strains Δ*arcA *and Δ*iclR*, and the double knockout strain Δ*arcA*Δ*iclR *cultivated in glucose abundant (batch) and glucose limiting (continuous) conditions.** The ratios, shown in Figure 4, were used as constraints to determine net fluxes. From top to bottom, values represent fluxes of the wild type, the Δ*arcA *and Δ*iclR *strain, and the Δ*arcA*Δ*iclR *strain. Standard errors are calculated by propagating measured errors of extracellular fluxes and ratios. Absolute fluxes in  were rescaled to the glucose uptake rate (shown in the upper boxes) to allow a clear comparison in flux distribution between the different strains.

Making use of the determined flux ratios as constraints in a stoichiometric model with known extracellular fluxes, the intracellular fluxes can be determined. To allow a clear comparison in flux distribution between the different strains, absolute fluxes in  were rescaled to the glucose uptake rate and the resulting metabolic fluxes are depicted in Figure 5.

Under glucose abundant conditions (Figure 5A) the Δ*arcA *strain exhibits a significantly higher TCA flux as opposed to the wild type. This is the result of the omission of repression due to *arcA *deletion on transcription of almost all TCA cycle genes or operons: *gltA*, *acnAB*, *icd*, *sucABCD*, *lpdA*, *sdhCDAB*, *fumAC*, and *mdh *[10,50-53] which was also observed by [15]. This further demonstrates the regulatory action of ArcA under aerobic conditions, although its main action was considered to be under microaerobic growth conditions [13,14]. The *iclR *single knockout strain exhibits similar glycolytic fluxes compared to the wild type, but at the PEP-pyruvate-oxaloacetate node fluxes are profoundly altered. Due to the *iclR *deletion, transcription of glyoxylate pathway genes is not longer inhibited. The flux data are in line with the isocitrate lyase activity measurements as shown in Table 2. In the Δ*iclR *and the Δ*arcA*Δ*iclR *strain the activation of the glyxoylate pathway is linked to only a minor increase in the flux from oxaloacetate to PEP, implying that the PEP-glyxoylate cycle is not active under glucose excess. As a result, part of the carbon is channeled through the glyoxylate pathway, less CO_2 _is produced in the TCA cycle and the extra CO_2 _saved is not lost in the oxaloacetate to PEP reaction, contributing to the higher biomass yield observed in these strains. This corresponds with the lower CO_2 _yields of these strains in Figure 1A.

Under glucose limitation, relative fluxes around the PEP-pyruvate-oxaloacetate node are higher as opposed to under glucose excess. Not only the flux converting pyruvate to acetyl-CoA at the entrance of the TCA cycle is increased, but also the glyoxylate pathway is active and gluconeogenic fluxes from malate to pyruvate and from oxaloacetate to PEP are higher compared to under batch conditions. These reactions create the PEP-glyoxylate cycle. This novel metabolic cycle was identified quite recently [21] and functions as an alternative to the TCA cycle for the oxidation of carbohydrates. Similar to the TCA cycle, this pathway produces CO_2_, *i.e. *in the reaction from OAA to PEP. As a result of the simultaneous activity of the TCA cycle and the PEP-glyoxylate cycle, more glucose is oxidized to CO_2 _compared to batch cultures in order to produce energy and meet the higher maintenance demand [36]. This is in accordance with the higher CO_2 _production and O_2 _consumption observed in glucose limited cultures (see Figure 1B vs 1A). Another effect observed between glucose limiting and abundant growth conditions is the reduced flux from 6-phosphogluconate to pentose-5-P by 6-phosphogluconate dehydrogenase (Gnd) for all strains in glucose limiting conditions (see Figure 5B vs 5A), which could be explained by the reduced transcription of *gnd *at lower growth rates [54-56].

### Glyoxylate pathway flux data and regulation of the *aceBAK *operon

The glyoxylate pathway flux data can also be used to investigate the interplay of different regulators on the *aceBAK *operon.

Under batch conditions, when Crp-cAMP levels are low and Crp cannot perform its activating role, no *aceBAK *transcription occurs and the glyoxylate pathway is inactive. However when the *aceBAK *repressor IclR is absent (*i.e. *in the Δ*iclR *strain), the glyoxylate pathway is active. This is illustrated by calculating the AceA/(AceA + Icd) flux ratio, which is much higher in the Δ*iclR *strain (32%) compared to the wild type (0%). This shows that Crp activation is not absolutely necessary for transcription. The absence of the repressor IclR is sufficient to obtain glyoxylate pathway activity. On the contrary, under glucose limitation, Crp-cAMP levels are high [2], the *aceBAK *transcription is enhanced and the glyoxylate bypass is active even in the presence of the repressor IclR. This is in line with the high value of the AceA/(AceA + Icd) flux ratio of the wild type (55%) compared to under batch conditions (0%). If under glucose limiting conditions *iclR *is inactivated, the AceA/(AceA + Icd) flux ratio increases even further to 63%. This clearly shows that both Crp and IclR regulate the *aceBAK *operon independently.

Under glucose abundant conditions, deleting *arcA *does not have a major effect on glyoxylate pathway fluxes (wild type vs. Δ*arcA *and Δ*iclR *vs. Δ*arcA*Δ*iclR*), despite the fact that ArcA is a known repressor of the *aceBAK *operon [57]. This is in stark contrast with the glyoxylate pathway fluxes under glucose limiting conditions. Here, *arcA *deletion reduces the bypass activity but only in a Δ*iclR *genetic environment. This is illustrated by the AceA/(AceA + Icd) flux ratio, which decreases from 55% in the wild type to 34% in the Δ*arcA*Δ*iclR *strain). However, the regulatory mechanism behind this remains unclear and needs to be resolved. Compared to the wild type, the Δ*arcA *strain has a similar overall flux distribution which was also found by Nanchen *et al. *[23], but contradicts the data obtained by Nizam *et al. *[58]

### Physiological comparison between *E. coli *K12 ΔarcAΔiclR and *E. coli *BL21

As explained in the previous sections the double knockout strain *E. coli *K12 Δ*arcA*Δ*iclR *shows an improved formation of biomass under both glucose abundant and limiting conditions (see Figure 1), with the most distinct effect under glucose abundant conditions (50% increase). This is mainly attributed to a reduced acetate and CO_2 _formation. After investigation of the intracellular fluxes (Figure 5A), the higher biomass yield under batch conditions can be explained by the activity of the glyoxylate pathway and the concomitant lower CO_2 _loss in the TCA. Furthermore, as a result of *arcA *deletion, repression on TCA cycle genes is removed, resulting in a higher TCA flux and a lower acetate formation. Also a slight increase in glycogen content was noticed in this strain under both growth conditions as shown in Table 3.

**Figure 6:**
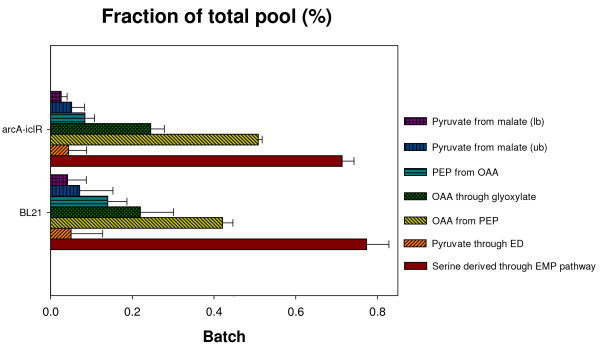
**Comparison of origin of metabolic intermediates in *E. coli *MG1655 Δ*arcA *Δ*iclR *and *E. coli *BL21 (DE3) under glucose abundant conditions**. Standard deviations are calculated on different samples originating from different cultivations. The serine through EMP and the pyruvate through ED results were obtained from experiments using 50% 1-^13^C glucose and 50% naturally labeled glucose. To determine the remaining values a mixture of 20% U-^13^C glucose and 0 naturally labeled glucose was used. To determine the fractions resulting in the formation of OAA a Monte-Carlo approach was applied.

**Figure 7:**
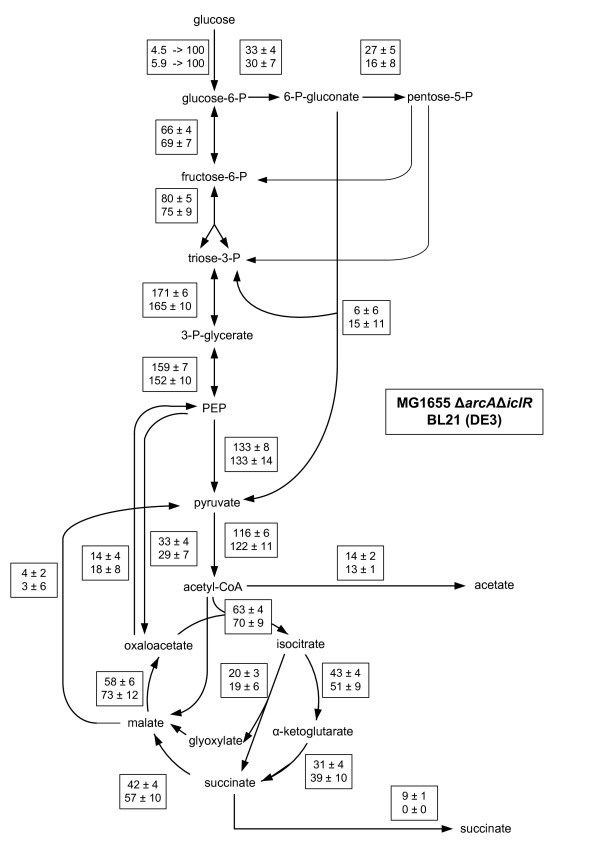
**Comparison of metabolic flux distribution in *E. coli *MG1655 Δ*arcA*Δ*iclR *and *E. coli *BL21 (DE3) cultivated under glucose abundant conditions**. The ratios, shown in Figure 6, were used as constraints to determine net fluxes. Standard errors are calculated by propagating measured errors of extracellular fluxes and ratios. Absolute fluxes in  were rescaled to the glucose uptake rate (shown in the upper boxes) to allow a clear comparison in flux distribution between the different strains.

Many of these characteristics are also attributed to *E. coli *BL21 (DE3) and therefore metabolic flux ratios and netto fluxes were determined for this strain as well and compared with *E. coli *K12 Δ*arcA*Δ*iclR *as illustrated in Figure 6 and 7, respectively. Small differences are observed in the OAA from PEP fraction, but this does not seem to influence the metabolic fluxes profoundly as almost all fluxes do not significantly differ between the two strains.

**Figure 8:**
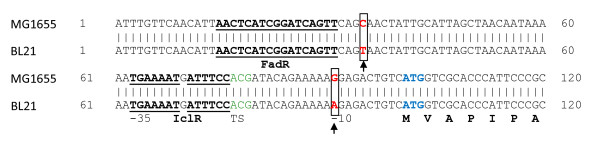
**BLAST analysis of the *iclR *promoter**. Basic Local Alignment Search of the promoter region of *iclR *in an *E. coli *K12 MG1655 and BL21 reveals 2 mutations (highlighted by boxes) in the BL21 strain. The binding sites of the regulators FadR and IclR (autoregulator) are underlined. TS stands for transcription start. Results were obtained using the NCBI online tool http://blast.ncbi.nlm.nih.gov.

A possible hypothesis is the following. Microarray data and Northern blot analysis showed that genes coding for enzymes participating in reactions involving gluconeogenesis, the TCA cycle and glycogen biosynthesis were upregulated in *E. coli *BL21 compared to *E. coli *K12 [59]. The higher *aceA *and *aceB *transcription in BL21 is caused by the apparent lower transcription of the *iclR *repressor [60]. Consequently, lower IclR levels are present in the cell and the glyoxylate pathway is active [61]. The lower transcription of *iclR *in *E. coli *BL21 may be explained by two mutations in the *iclR *promoter region compared to *E. coli *K12 MG1655 (BLAST analysis, Figure 8). Particularly the mutation close to the Pribnow box or -10 box is important as it can have a major effect on the binding of RNA polymerase and hence gene expression [62,63].

Not only is the glyoxylate flux similar, the TCA flux is improved as well in both strains compared to the *E. coli *K12 MG1655 wild type. Release of repression on transcription of TCA genes explains the higher flux in *E. coli *K12 Δ*arcA*Δ*iclR *[10], and this must also be valid for *E. coli *BL21 as transcription of its TCA genes was highly upregulated compared to *E. coli *K12 [59]. Genome comparison showed that although BL21 and K12 genomes align for > 99%, many minor differences appear, which can explain the metabolic differences observed [64,65]. However, those studies did not focus on differences in *arcA *regions. Using a Basic Local Alignment Search Tool (BLAST) it was determined that there is a 99% similarity in the *arcA *gene between the two strains. Only five minor mutations are observed (BLAST results shown in Additional file 3). However, the consequence of these mutations is that five other codons are formed in the mRNA in BL21 as opposed to MG1655 (see Table 4). These different codons in BL21 still encrypt for the same amino acids but two of these five codons (*i.e. *CUA and UCC) are known low-usage codons in *E. coli *and can cause translational problems [66,67]. Therefore it is likely that due to a different codon usage in BL21, *arcA *activity is decreased, which can explain the similar higher TCA flux observed between the two strains.

**Table 4:** Comparison of the codon usage in the *arcA *gene between *E. coli *K12 MG1655 and BL21 (DE3) based on Chen & Texada, [66].

AA	Strain	Codon	Frequency	tRNA content
L	MG1655	CUG	54.1	1
	BL21	CUA	2.97	Minor
				
S	MG1655	UCU	10.47	0.25
	BL21	UCC	9.43	Minor
				
P	MG1655	CCA	8.12	Major
	BL21	CCG	23.91	Major
				
I	MG1655	AUC	26.97	1
	BL21	AUU	27.27	1
				
C	MG1655	UGU	4.8	Minor
	BL21	UGC	6.07	Minor

Each codon is expressed as the frequency per 1000 codons. The content is the relative amount to that of tRNALeu1(CUG), which is normalized to 1 and approximately in the order of 104 molecules per cell for normally growing *E. coli *cells

## Conclusions

Under glucose abundant conditions the double knockout strain *E. coli *MG1655 Δ*arcA*Δ*iclR *exhibits an increased biomass yield of 0.63 c-mole/c-mole glucose, which approximates the maximum theoretical yield of 0.65 c-mole/c-mole glucose. Also under glucose limitation a higher biomass yield was observed, but effects were less distinct due to a fixed growth rate and a higher maintenance. The higher biomass formation is accompanied by a decrease in acetate formation and CO_2 _production. Only a small part of the higher yield was attributed to an increased glycogen content. Furthermore, enzyme activity measurements showed an increased transcription of glyoxylate enzymes, implying the activation of this pathway in the Δ*arcA*Δ*iclR *strain even under glucose abundant conditions, when Crp-activation is absent. This was confirmed by ^13^*C *metabolic flux analysis, showing that 30% of isocitrate molecules were channeled through the glyoxylate pathway when *iclR *was knocked out. Deletion of *arcA *results in loss of repression on transcription of TCA genes, which provokes a higher flux through the TCA cycle. This explains the lower acetate formation observed. Because many physiological and metabolic properties observed in the double knockout strains are also attributed to *E. coli *BL21, the metabolic fluxes of the two strains were compared under glucose abundant conditions. Almost all fluxes in central metabolism seemed to be similar, which can be explained by mutations in the promoter region of *iclR *and a less efficient codon usage of *arcA *in BL21, resulting in lower activity of the corresponding enzymes.

## Methods

### Strains

The strains used in this study are listed in Table 5. *Escherichia coli *MG1655 [λ^-^, *F *^-^, *rph *-1] and BL21 were obtained from the Coli Genetic Stock Center (CGSC). The single and double knockout strains were constructed using a one-step disruption protocol [68]. In order to confirm the mutations, polymerase chain reaction (PCR) was used to amplify fragments containing the modified sequences. Lengths of amplified fragments were tested by agarose gel electrophoresis and compared with those of the wild type strain (WT). PCR products were also sequenced to confirm knockouts and sequence substitutions. The different strains were preserved in a (1:1) glycerol:LB growth medium solution.

**Table 5:** List of strains used.

Strain	Description	Reference
MG1655	wild type	Coli Genetic Stock Center
MG1655 Δ*arcA*	ArcA knockout strain	This study
MG1655 Δ*iclR*	IclR knockout strain	This study
MG1655 Δ*arcA*Δ*iclR*	ArcA-IclR double knockout strain	This study
BL21 (DE3)	wild type	Coli Genetic Stock Center

### Media

Luria Broth (LB) medium consisted of 10 *g*.*L *^-1 ^tryptone peptone (Difco, Belgium), 5 *g*.*L *^-1 ^yeast extract (Difco) and 10 *g*.L ^-1 ^sodium chloride. Shake flask medium (S) contained 2 *g*.*L *^-1 ^NH_4_Cl, 5 *g*.*L *^-1 ^(NH_4_)_2_SO_4_, 2.993 *g*.*L *^-1 ^KH_2_PO_4_, 7.315 *g*.*L *^-1 ^K_2_HPO_4_, 8.372 *g*. *L *^-1 ^MOPS, 0.5 *g*. *L *^-1 ^NaCl, 0.5 *g*.*L *^-1 ^MgSO_4 _· 7 H_2_O, 16.5 *g*.*L *^-1 ^glucose · H_2_O, 1 *mL*.*L *^-1 ^trace element solution and 100 *μL*.*L *^-1 ^molybdate solution. The medium was set to a pH of 7 with 1 M KH_2_PO_4_.

The minimal medium during fermentations (M1) in a benchtop bioreactor contained 6.75 *g*.*L*^-1 ^NH_4_Cl, 1.25 *g*.*L *^-1 ^(NH_4_)_2_SO_4_, 1.15 *g*.*L *^-1 ^KH_2_PO_4_, 0.5 *g*.*L *^-1 ^NaCl, 0.5 *g*.*L *^-1 ^MgSO_4 _· 7 H_2_O, 16.5 *g*.*L *^-1 ^glucose · H_2_O, 1 *mL*.*L *^-1 ^trace element solution and 100 *μL*.*L *^-1 ^molybdate solution.

In ^13^C-flux analysis experiments, minimal medium for minireactors (M2) was used. This medium contained 1 *g*.*L *^-1 ^NH_4_Cl, 1 *g*.*L *^-1 ^(NH_4_)_2_SO_4_, 3 *g*.*L *^-1 ^KH_2_PO_4_, 7.315 *g*.*L *^-1 ^Na_2_HPO_4_, 0.5 *g*.*L *^-1 ^NaCl, 0.5 *g*.*L *^-1 ^MgSO_4 _· 7 H_2_O, 3 *g*.*L *^-1 ^glucose, 1 *mL*.*L *^-1 ^trace element solution, 100 *μL*.*L *^-1 ^molybdate solution. The glucose used in this M2 medium was added as a mixture of 20% U-^13^C glucose (99% purity) and 80% naturally labeled glucose or as a mixture of 50% 1-^13^C glucose (99% purity) and 50% naturally labeled glucose depending on the flux ratios that needed to be identified. Trace element solution consisted of 3.6 *g*.*L *^-1 ^FeCl_2 _· 4 H_2_O, 5 *g*.*L *^-1 ^CaCl_2 _· 2 H_2_O, 1.3 *g*.*L *^-1 ^MnCl_2 _· 2 H_2_O, 0.38 *g*.*L *^-1 ^CuCl_2 _· 2 H_2_O, 0.5 *g*.*L *^-1 ^CoCl_2 _· 6 H_2_O, 0.94 *g*.*L *^-1 ^ZnCl_2_, 0.0311 *g*.*L *^-1 ^H_3_BO_4_, 0.4 *g*.*L *^-1 ^Na_2_EDTA · 2 H_2_O, 42 *g*.*L *^-1^SeO_2 _and 1.01 *g*.*L *^-1 ^thiamine · HCl. The molybdate solution contained 0.967 *g*.*L *^-1 ^Na_2_MoO_4 _· 2 H_2_O. If not specifically mentioned, all chemicals were purchased at Sigma, Belgium.

### Cultivation conditions

To determine substrate uptake and product secretion rates, enzyme activities, and glycogen and trehalose contents, cells were cultivated in 2*L *benchtop bioreactors, since higher volume vessels improve accuracy of the measurements. However, in order to map the metabolic fluxes in the cell, expensive ^13^C-labeled substrates are necessary and therefore alternative miniscale reactors were chosen as the method of cultivation. Earlier studies have shown that similar growth conditions were achieved in the benchtop and miniscale reactor setups [69,70].

For experiments in bioreactors, a preculture in a test tube filled with 5 *mL *LB medium was inoculated with a single colony from a LB-plate and incubated during 8 hours at 37°*C *on an orbital shaker at 200 *rpm*. From this culture, 2 *mL *was transferred to 100 *mL *minimal medium (S) in a 500 *mL *shake flask and incubated for 16 hours at 37°*C *on an orbital shaker at 200 *rpm*. A 4% inoculum was used in a 2*L *Biostat B Plus culture vessel with 1.5 L working volume (Sartorius Stedim Biotech, Germany). The culture conditions were: 37°*C*, stirring at 800 *rpm*, and a gas flow rate of 1.5 *L*.*min *^-1^. The pH was maintained at 7 with 0.5 M H_2_SO_4 _and 4 M KOH. The exhaust gas was cooled down to 4°*C *by an exhaust cooler (Frigomix 1000, Sartorius Stedim Biotech, Germany). A 10% solution of silicone antifoaming agent (BDH 331512K, VWR Int Ltd., England) was added when foaming increased during the fermentation (approximately 10 *μL*). The off-gas was measured with an EL3020 off-gas analyser (ABB Automation GmbH, Germany). All data were logged with the Sartorius MFCS/win v3.0 system (Sartorius Stedim Biotech, Germany). All strains were cultivated at least twice and the given standard deviations on yields and rates are based on at least 10 data points taken during the repeated experiments.

For labeling experiments miniscale reactorsetups had to be used due to the high cost of the labeled substrate. Batch conditions were achieved in 24 deepwell microtiterplates [71], while continuous conditions were gained by using a bubblecolumn reactor [72]. In both cases an exponentially growing shake flask culture was used to inoculate minimal medium M2 to achieve an initial optical density (OD_595 *nm*_) of 0.02 in each well of the microtiterplate or each bubblecolumn reactor by varying the inoculation volume. 24 square deepwell plates (Enzyscreen, The Netherlands) were filled with 3 *mL *of M2 medium and were incubated at 37°*C *on an orbital shaker at 250 *rpm *(shaking diameter = 5 cm). Plates were closed with so called sandwich covers (Enzyscreen, The Netherlands) to prevent cross-contamination and evaporation. To further reduce evaporation, a shake flask filled with water was placed in the incubator. All strains were cultivated in at least twelvefold and in at least two different plates. The setup of the bubblecolumn reactor is described in more detail elsewhere [72]. The working volume was 10 *mL*. After the batch phase was completed, a dilution rate of 0.1 *h *^-1 ^was established.

### Sampling methodology

In batch cultivations, samples were taken during the exponential growth phase. In continuous experiments, samples were taken after at least 7 dilution times. The sampling method was the same as earlier described [69]. Glucose abundant conditions imply a glucose concentration higher than 5 *g*.*L *^-1 ^in the benchtop reactor experiments (15 *g*.*L *^-1 ^glucose in M1 medium) or higher than 1.5 *g*.*L *^-1 ^in the miniscale reactor setup experiments (3 *g*.*L *^-1 ^glucose in M2 medium). In batch experiments, glucose concentrations were never lower than 1 *g*.*L *^-1 ^in the samples used for comparative analysis. This concentration is more than 15 times higher than the glucose concentration of 54 *mg*.*L *^-1 ^at which an effect on cAMP levels (a marker of glucose limitation) can be noticed [73]. Glucose limiting conditions imply a glucose concentration lower than 5 *mg*.*L *^-1 ^[74]. Samples for enzyme activity measurements or metabolic flux analysis were always taken during the mid-exponential growth phase when the glucose concentration was not limiting growth.

### Determination of biomass, organic acids and glucose concentrations

The biomass content was obtained by centrifugation and subsequent drying of 20 *mL *reactor broth. The concentrations of glucose and organic acids were determined on a Varian Prostar HPLC system (Varian, Belgium), using an Aminex HPX-87H column (Bio-Rad, Belgium) heated at 65°*C*, equipped with a 1 *cm *reversed phase precolumn, using 5 *mM *H_2_SO_4 _(0.6 *mL*.*min *^-1^) as mobile phase. Detection and identification were performed by a dual-wave UV-VIS (210 *nm *and 265 *nm*) detector (Varian Prostar 325) and a differential refractive index detector (Merck LaChrom L-7490, Merck, Belgium). Metabolites detectable by HPLC were acetate, acetaldehyde, acetoin, ethanol, formate, fumarate, oxaloacetate, lactate, pyruvate, succinate and glucose. Product yields and (specific) product secretion rates were calculated based on end sample concentrations and maximum growth rate for MTPs and on concentrations of ten samples taken at different time points for benchtop bioreactors [70].

### Glycogen and trehalose content

Glycogen and trehalose assays were based on the method described by Parrou *et al. *[75]. In short, isoamylase, amyloglucosidase and trehalase (Sigma, Belgium) were used to degrade glycogen and trehalose to glucose. The glucose that is formed in these reactions was measured with a glucose oxidase peroxidase assay (GOD-POD). Standards were used to determine the glycogen and trehalose recovery (measured as 91% and 86%, respectively). Matrix effects were excluded by applying standard addition.

### Enzyme activity assays for malate synthase and isocitrate lyase

Samples for these measurements were kept at 80°*C *until analysis. A predetermined amount of cells was lyzed with the EasyLyse™ cell lysis kit (Epicentre Biotechnologies, The Netherlands) and the cell extract was kept at 4°*C *Isocitrate lyase assay was adopted from [76]. This colorimetric method is based on the reaction of glyoxylate, a product of isocitrate lyase, with phenylhydrazine. The reaction mixture is composed of 6 *mM *magnesium chloride, 4 mM phenylhydrazine, 12 *mM *L-cystein, and 8 *mM *trisodium isocitrate in a 100 *mM *potassium phosphate buffer (pH 7). 985 L of this mixture was added to 15 *μL *of enzyme extract. Enzyme activity was measured at 324 *nm *at 30°*C *(Uvikom 922 spectrophotometer, BRS, Belgium). The malate synthase assay was also adopted from [76]. This is a colorimetric assay based on the reaction of coenzyme CoA with DTNB (5,5'-dithio-bis-(2-nitrobenzoate)). The reaction mixture of this assay is composed of 15 mM magnesium chloride, 0.2 mM acetyl-CoA, 10 mM glyoxylate and 0.2 *mM *DTNB in a 100 *mM *Tris buffer (pH 8). 900 *μL *of this mixture was added to 100 *μL *enzyme extract. The enzyme activity was measured at 412 *nm *at 30°*C*. The activity was normalized to the amount of biomass used for the assay and is expressed in *μ*mol per minute per gram biomass.

### GC-MS analysis of amino acids

The analysis of the isotopic labeling of amino acids was based on [77]. Briefly, cell pellets, sampled at steady state (*OD*_595 _= ±1) were hydrolyzed with 6*M *HCl at 105°*C *for 24 *h *in sealed eppendorf tubes. Subsequently the hydrolyzates were dried in a Thermomixer (Eppendorf, VWR, Belgium) at 90°*C *for no longer than 12 *h*. Amino acids were extracted from the hydrolyzed pellet using 30 *μL *dimethylformamide (Acros Organics, Belgium) and derivatized with 30 *μ*L

N-(tert-butyldimethylsilyl)-N-methyltrifluoroacetamide (MTBSTFA) + 1% tert-butyldimethylchlorosilane (TBDMSCl) (Sigma, Belgium) for 1 h at 85°*C*. 1 *μL *of this mixture was injected into a TRACE gas chromatograph connected to a DSQ mass spectrometer (Thermo, Interscience, Belgium) equipped with a TR-1 (30 *m *× 0.25 *mm *× 0.25 *μm*, Thermo) column. The carrier gas was helium and the flow was set at 1.5 *ml*.*min *^-1 ^with flow mode in split control (split ratio 10.1). The oven temperature was initially kept at 160°*C *for 1 min and then the temperature was gradually increased to 310°*C *at a rate of 20°C.*min *^-1 ^The final temperature was kept for 0.5 *min*. The injector and the ion source temperature were set at 230°*C*. Electron impact ionization was performed at 70*eV *. Mass spectra were analyzed in full scan mode from 180 to 550 amu's with a scan rate of 1400 *amu*.*s*^-1^. The obtained mass distribution vectors of the fragments of the amino acids were corrected for naturally occurring isotopes [78].

### ^13^C-Constrained metabolic flux analysis

^13^C-Flux analysis was based on the calculation of metabolic ratios and consequently using these ratios as constraints in net flux analysis [78]. In short, based upon the corrected mass distribution vectors of the proteinogenic amino acids the ^13^C-labeling patterns of central metabolites were calculated. Using this labeling information, metabolic flux ratios could be calculated using the software FiatFlux [79]. Since the calculation of the ratio of OAA molecules originating from PEP, the glyoxylate shunt, or the TCA shunt is not present in the official FiatFlux release, a new Matlab program had to be written using a slightly corrected version of the equation presented by Nanchen *et al. *[72]:(1)

where *f*_1_, *f*_2 _and (1 - *f*_1 _- *f*_2_) resemble the fractions of OAA molecules originating from anaplerosis, the glyoxylate shunt, and the TCA cycle, respectively. The labeling of a molecule *X *in this equations is expressed as *X*_*a*-*b *_where _*a*-*b *_indicates the carbon atoms considered. *C*_1 _is a one carbon atom with the fractional labeling of the input substrate.

To solve this equation, a Monte-Carlo approach was implemented in Matlab. First, average mass distribution vectors (mdv's) and standard deviations for every *X*_*a*-*b *_were calculated based upon at least 10 GC-MS analyses of different biological samples. Next, samples were taken in the mdv measurement matrix using the normrnd function. Finally, the equation was solved by varying *f*_1_, *f*_2 _and the fractional labeling of *CO*_2 _and the best fit solution was kept. Step 2 and 3 of this calculation process were repeated 1000 times and all values of *f*_1_, *f*_2_, and the measured labeling of *CO*_2 _were plotted to check if the parameters were normally distributed. If this was valid, average values and standard deviations for these parameters were calculated.

Subsequently, intracellular fluxes were calculated in the NETTO module of Fiatflux, using a slightly modified version of a previously described stoichiometric model [70], extended with succinate transport out of the cell. This model consisted in total of 27 reactions and 22 balanced metabolites. Glucose uptake, succinate and acetate excretion were experimentally determined. The effluxes of precursor metabolites to biomass formation was estimated based on the growth rate dependent biomass composition of *E. coli *[80-82]. The underdetermined system of equations with 5 degrees of freedom was solved by using the following 7 ratios as constraints: Serine from glycolysis, Pyruvate through ED pathway, Pyruvate from malate (upper and lower bound), OAA originating from PEP, OAA originating from glyoxylate, and PEP originating from OAA.

## Authors' contributions

HW and HM performed ^13^C-labeling experiments, HPLC and GC-MS analyses and flux analysis. JB performed the benchtop bioreactor experiments and corresponding HPLC analyses and enzyme assays. MFM constructed the knock-out strains. HW and JB drafted the manuscript. JM revised the manuscript critically. All authors read and approved the final manuscript.

## Supplementary Material

Additional file 1 **Average carbon and redox balances for batch and chemostat cultures**. This file may be accessed using Microsof Excel or OpenOffice Spreadsheet. Click here for file

Additional file 2 **Corresponding gene products of genes used in Figure **2. This file may be accessed using Microsof Word or OpenOffice Word Processor. Click here for file

Additional file 3 **BLAST analysis of the *arcA *gene**. This file may be accessed using Microsof Word or OpenOffice Word Processor. Click here for file
