# Supplementary material for: Effect of iclR and arcA knockouts on biomass formation and metabolic fluxes in Escherichia coli K12 and its implications on understanding the metabolism of Escherichia coli BL21 (DE3)
Source: BMC Microbiol. 2011 Apr 11;11:70. doi: 10.1186/1471-2180-11-70 (PMC3094197; doi:10.1186/1471-2180-11-70)
Supplement: Additional file 2 — Corresponding gene products of genes used in Figure 2. This file may be accessed using Microsof Word or OpenOffice Word Processor. [file 1471-2180-11-70-S2.DOC]

## Genes and gene products

| Gene | Gene product |
| --- | --- |
| *aceA* | Isocitratelyase |
| *aceB* | Malate synthase |
| *ackA* | Acetate kinase |
| *acn* | Aconitase |
| *acs* | AcetylCo synthase |
| *adhCEP* | Ethanol dehydrogenase |
| *adhE* | Acetyldehydrogenase |
| *aldH* | Aldehyde dehydrogenase |
| *alsI* | Ribose-5-phosphate isomerase B / allose-6-phosphate isomerase |
| *aspA* | Aspartate ammonia-lyase |
| *aspC* | Aspartate aminotransferase |
| *citDEF* | Citrate lyase |
| *dctA* | Succinate importer |
| *dcuC* | Succinate exporter |
| *eda* | Oxaloacetate decarboxylase / 2-keto-3-deoxy-6-phosphogluconate aldolase ,/ 2-keto-4-hydroxyglutarate aldolase |
| *edd* | Phosphogluconate dehydratase |
| *eno* | Enolase |
| *fbaAB* | Fructosebisphosphate aldolase |
| *fbp* | Fructose-1,6-bisphosphatase I |
| *focA, focB* | Formate FNT transporter |
| *fumABCD* | Fumarase |
| *gapA* | Glyceraldehyde-3-phosphate dehydrogenase |
| *glk* | PTS, glucokinase |
| *glpX* | Fructose-1,6-bisphosphatase II |
| *gltA* | Citrate synthase |
| *gnd* | 6-phosphogluconate dehydrogenase |
| *gpmAM, ytjC* | Phophoglyceratemutase |
| *icd* | Isocitrate dehydrogenase |
| *ilvBG_2_HIMN* | Acetolacetate decarboxylase |
| *ldhA* | Lactate dehydrogenase |
| *lldP* | Lactate transporter |
| *maeA,B* | Malic enzyme |
| *mdh* | Malate dehydrogenase |
| *mgsA* | Methylglyoxal synthase |
| *pck* | Phosphoenolpyruvatecarboxykinase |
| *pdh* | Pyruvate dehydrogenase |
| *pfkA,B* | 6-phophofructokinase |
| *pflB, tdcE* | Pyruvateformatelyase |
| *pgi* | Phosphogluco-isomerase |
| *pgi* | Phosphoglucose isomerase |
| *pgk* | Phophoglyceratekinase |
| *poxB* | Pyruvate oxidase |
| *ppc* | Phosphoenolpyruvatecarboxylase |
| *pps* | Phosphoenolpyruvate synthase |
| *pta* | Acetylphosphotransferase |
| Gene | Gene product |
| *pyk* | Pyruvate kinase |
| *rpe* | Ribose-5-phosphate epimerase |
| *rpiA* | Ribose-5-phosphate isomerase A |
| *sdhABCD* | Succinate dehydrogenase |
| *sucAB,lpd* | α-ketoglutarate dehydrogenase |
| *sucCD* | Succinate thiokinase |
| *talA, talB* | Transaldolase A; transaldolase B |
| *tktA, tktB* | Transketolase I; transketolase II |
| *tpi* | Triosephosphateisomerase |
| *ybhA* | Pyridoxalphosphatase| Fructose-1,6-bisphosphatase |
| *zwf* | Glucose-6-phosphate-1-dehydrogenase |
