# Supplementary material for: Effect of iclR and arcA knockouts on biomass formation and metabolic fluxes in Escherichia coli K12 and its implications on understanding the metabolism of Escherichia coli BL21 (DE3)
Source: BMC Microbiol. 2011 Apr 11;11:70. doi: 10.1186/1471-2180-11-70 (PMC3094197; doi:10.1186/1471-2180-11-70)
Supplement: Additional file 3 — BLAST analysis of the arcA gene. This file may be accessed using Microsof Word or OpenOffice Word Processor. [file 1471-2180-11-70-S3.DOC]

ArcA

A: MG1655

B: BL21

Score = 2307 bits (2558), Expect = 0.0

Identities = 1290/1297 (99%), Gaps = 0/1297 (0%)

Strand=Plus/Plus

A 1 TACGCGGTGCGAATTTACAAAT**C**CTTAACGTAAGTCGCAGAAAAAG**CCCTTTACT**TAGCT 60

|||||||||||||||||||||| |||||||||||||||||||||||||||||||||||||

B 1 TACGCGGTGCGAATTTACAAAT**T**CTTAACGTAAGTCGCAGAAAAAG**CCCTTTACT**TAGCT 60

-35(7)

A 61 TAAAAAAG**GCTAAACT**ATTTCCTGA**CTG**TACTAACGGTTGA**A**TTGTTAAAAAATGCTACA 120

||||||||||||||||||||||||||||||||||||||||| ||||||||||||||||||

B 61 TAAAAAAG**GCTAAACT**ATTTCCTGA**CTG**TACTAACGGTTGA**G**TTGTTAAAAAATGCTACA 120

-10(7) TS(7)

A 121 TATCCTTCTGTTTACTTAGGATAATTTTATAAAAAATAAATC**TCGACA**AT*TGGATTCA*CC 180

||||||||||||||||||||||||||||||||||||||||||||||||||||||||||||

B 121 TATCCTTCTGTTTACTTAGGATAATTTTATAAAAAATAAATC**TCGACA**AT*TGGATTCAC*C 180

-35(6) *-35(5)*

A 181 ACGTTTAT**TAGT*T****GTATG****AT*G**CAAC**TAG**TTGGATTATTAAAATAATGTGACGAAAGCTAG 240

||||||||||||||||||||||||||||||||||||||||||||||||||||||||||||

B 181 ACGTTTAT**TAGT*T****GTATG****AT*G**CAAC**TAG**TTGGATTATTAAAATAATGTGACGAAAGCTAG 240

-10(6)*-10(5)*TS(6) *TS(5)*

A 241 CATTT**AGATACGAT**GATTTCATCAAAC**TGTTAACGT**GCTACAATTGA**ACTTGATATATGT** 300

||||||||||||||||||||||||||||||||||||||||||||||||||||||||||||

B 241 CATTT**AGATACGAT**GATTTCATCAAAC**TGTTAACGT**GCTACAATTGA**ACTTGATATATGT**  300

-35(4) -10(4) TS(4) **FNR**

A 301 **CAA**CGAAGCGTAGTTTTATT**GGGTGTCCG**GCCCCTCTTAGC*C****TGTTATGT***T**GCT**GTTAAA 360

||||||||||||||||||||||||||||||||||||||||||||||||||||||||||||

B 301 **CAA**CGAAGCGTAGTTTTATT**GGGTGTCCG**GCCCCTCTTAGC*C****TGTTATGT***T**GCT**GTTAAA 360

-35(3) -35(2)(1)-10(3)TS(3)

A 361 AT**GGTTAGGAT**GAC**AGC**C**GTT**TTTGACACTGTCGGGTCCTGAGGGAAAGTACCCACGACC 420

||||||||||||||||||||||||||||||||||||||||||||||||||||||||||||

B 361 AT**GGTTAGGAT**GAC**AGC**C**GTT**TTTGACACTGTCGGGTCCTGAGGGAAAGTACCCACGACC 420

-10(2)(1) TS(2)TS(1)

A 421 AAGCTAATGATGTTGTTGACGTTGATGGAAAGTGCATCAAGAACGCAATTACGTACTTTA 480

||||||||||||||||||||||||||||||||||||||||||||||||||||||||||||

B 421 AAGCTAATGATGTTGTTGACGTTGATGGAAAGTGCATCAAGAACGCAATTACGTACTTTA 480

A 481 GTCATGTTACGCCGATCATGTTAATTTGCAGCATGCATCAGGCAGGTCAGGGACTTTTGT 540

||||||||||||||||||||||||||||||||||||||||||||||||||||||||||||

B 481 GTCATGTTACGCCGATCATGTTAATTTGCAGCATGCATCAGGCAGGTCAGGGACTTTTGT 540

A 541 ACTTCCTGTTTCGATTTAGTTGGCAATTTAGGTAGCAAAC**ATG**CAGACCCCGCACATTCT 600

||||||||||||||||||||||||||||||||||||||||||||||||||||||||||||

B 541 ACTTCCTGTTTCGATTTAGTTGGCAATTTAGGTAGCAAAC**ATG**CAGACCCCGCACATTCT 600

**M Q T P H I L**

A 601 TATCGTTGAAGACGAGTTGGTAACACGCAACACGTTGAAAAGTATTTTCGAAGCGGAAGG 660

||||||||||||||||||||||||||||||||||||||||||||||||||||||||||||

B 601 TATCGTTGAAGACGAGTTGGTAACACGCAACACGTTGAAAAGTATTTTCGAAGCGGAAGG 660

**I V E D E L V T R N T L K S I F E A E G**

A 661 CTATGATGTTTTCGAAGCGACAGATGGCGCGGAAATGCATCAGATCCTCTCTGAATATGA 720

||||||||||||||||||||||||||||||||||||||||||||||||||||||||||||

B 661 CTATGATGTTTTCGAAGCGACAGATGGCGCGGAAATGCATCAGATCCTCTCTGAATATGA 720

**Y D V F E A T D G A E M H Q I L S E Y D**

A 721 CATCAACCTGGTGATCATGGATATCAATCTGCCGGGTAAGAACGGTCTTCTGTTAGCGCG 780

||||||||||||||||||||||||||||||||||||||||||||||||||||||||||||

B 721 CATCAACCTGGTGATCATGGATATCAATCTGCCGGGTAAGAACGGTCTTCTGTTAGCGCG 780

**I N L V I M D I N L P G K N G L L L A R**

A 781 TGAACTGCGCGAGCAGGCGAATGTTGCGTTGATGTTCCTGACTGGCCGTGACAACGAAGT 840

||||||||||||||||||||||||||||||||||||||||||||||||||||||||||||

B 781 TGAACTGCGCGAGCAGGCGAATGTTGCGTTGATGTTCCTGACTGGCCGTGACAACGAAGT 840

**E L R E Q A N V A L M F L T G R D N E V**

A 841 CGATAAAATTCTCGGCCTCGAAATCGGTGCAGATGACTACATCACCAAACCGTTCAACCC 900

||||||||||||||||||||||||||||||||||||||||||||||||||||||||||||

B 841 CGATAAAATTCTCGGCCTCGAAATCGGTGCAGATGACTACATCACCAAACCGTTCAACCC 900

**D K I L G L E I G A D D Y I T K P F N P**

A 901 GCGTGAACTGACGATTCGTGCACGCAACCT**G**CTGTCCCGTACCATGAATCTGGGTACTGT 960

|||||||||||||||||||||||||||||| |||||||||||||||||||||||||||||

B 901 GCGTGAACTGACGATTCGTGCACGCAACCT**A**CTGTCCCGTACCATGAATCTGGGTACTGT 960

**R E L T I R A R N L L S R T M N L G T V**

A 961 CAGCGAAGAACGTCGTAGCGTTGAAAGCTACAAGTTCAATGGTTGGGAACTGGACATCAA 1020

||||||||||||||||||||||||||||||||||||||||||||||||||||||||||||

B 961 CAGCGAAGAACGTCGTAGCGTTGAAAGCTACAAGTTCAATGGTTGGGAACTGGACATCAA 1020

**S E E R R S V E S Y K F N G W E L D I N**

A 1021 CAGCCGTTCGTTGATCGGCCCTGATGGCGAGCAGTACAAGCTGCCGCGCAGCGAGTTCCG 1080

||||||||||||||||||||||||||||||||||||||||||||||||||||||||||||

B 1021 CAGCCGTTCGTTGATCGGCCCTGATGGCGAGCAGTACAAGCTGCCGCGCAGCGAGTTCCG 1080

**S R S L I G P D G E Q Y K L P R S E F R**

A 1081 CGCCATGCTTCACTTCTGTGAAAACCCAGGCAAAATTCAGTC**T**CGTGCTGAACTGCTGAA 1140

|||||||||||||||||||||||||||||||||||||||||| |||||||||||||||||

B 1081 CGCCATGCTTCACTTCTGTGAAAACCCAGGCAAAATTCAGTC**C**CGTGCTGAACTGCTGAA 1140

**A M L H F C E N P G K I Q S R A E L L K**

A 1141 GAAAATGACCGGCCGTGAGCTGAAACC**A**CACGACCGTACTGTAGACGTGACGATCCGCCG 1200

||||||||||||||||||||||||||| ||||||||||||||||||||||||||||||||

B 1141 GAAAATGACCGGCCGTGAGCTGAAACC**G**CACGACCGTACTGTAGACGTGACGATCCGCCG 1200

**K M T G R E L K P H D R T V D V T I R R**

A 1201 TATTCGTAAACATTTCGAATCTACGCCGGATACGCCGGAAATCATCGCCACCAT**C**CACGG 1260

|||||||||||||||||||||||||||||||||||||||||||||||||||||| |||||

B 1201 TATTCGTAAACATTTCGAATCTACGCCGGATACGCCGGAAATCATCGCCACCAT**T**CACGG 1260

**I R K H F E S T P D T P E I I A T I H G**

A 1261 TGAAGGTTATCGCTTCTG**T**GGTGATCTGGAAGATTAA 1297

|||||||||||||||||| ||||||||||||||||||

B 1261 TGAAGGTTATCGCTTCTG**C**GGTGATCTGGAAGATTAA 1297

**E G Y R F C G D L E D STOP**
